# Crystal structure and Hirshfeld surface analysis of 2-{[7-acetyl-4-cyano-6-hy­droxy-8-(4-meth­oxy­phen­yl)-1,6-dimethyl-5,6,7,8-tetra­hydro­iso­quino­lin-3-yl]sulfan­yl}-*N*-phenyl­acetamide

**DOI:** 10.1107/S2056989021005430

**Published:** 2021-05-28

**Authors:** Eman M. Sayed, Reda Hassanien, Shaaban K. Mohamed, Joel T. Mague, Mehmet Akkurt, Nasser Farhan, Etify A. Bakhite, Safiyyah A. H. Al-Waleedy

**Affiliations:** a Chemistry Department, Faculty of Science, New Valley University, 72511 El-Kharja, Egypt; b Chemistry and Environmental Division, Manchester Metropolitan University, Manchester, M1 5GD, England; c Chemistry Department, Faculty of Science, Minia University, 61519 El-Minia, Egypt; d Department of Chemistry, Tulane University, New Orleans, LA 70118, USA; e Department of Physics, Faculty of Sciences, Erciyes University, 38039 Kayseri, Turkey; f Chemistry Department, Faculty of Science, Assiut University, 71516 Assiut, Egypt; g Chemistry Department, Faculty of Science, Taiz University, Taiz, Yemen

**Keywords:** crystal structure, tetra­hydro­iso­quinoline, hydrogen bond, C—H⋯π(ring) inter­action, amide

## Abstract

The title mol­ecule, C_29_H_29_N_3_O_4_S, adopts a conformation with the two phenyl substituents disposed on opposite sides of the mean plane of the iso­quinoline unit. In the crystal, corrugated layers of mol­ecules are formed by N—H⋯O, C—H⋯N and C—H⋯S hydrogen bonds together with C—H⋯π(ring) inter­actions. These layers are connected by C—H⋯O contacts. The Hirshfeld surface analysis of the crystal structure indicates that the most important contributions for the crystal packing are from H⋯H (45.2%), C⋯H/H⋯C (20.2%), O⋯H/H⋯O (15.8%) and N⋯H/H⋯N (11.0%) inter­actions.

## Abstract 2

The title mol­ecule adopts a conformation with the two phenyl substituents disposed on opposite sides of the mean plane of the iso­quinoline unit. In the crystal, corrugated layers of mol­ecules are formed by N—H⋯O, C—H⋯N and C—H⋯S hydrogen bonds together with C—H⋯π(ring) inter­actions. These layers are connected by C—H⋯O contacts.

## Chemical context   

Many tetra­hydro­iso­quinolines have medicinal importance as potent selective and orally active aldosterone synthase (CYP11B2) inhibitors (Martin *et al.*, 2016 ▸). There are many natural and modified natural products that contain annulated pyridine rings such as the fatty acid bending protein inhibitor, (−)-oxerine, (−)-actinidine, indicaine, and other compounds that are derived from flavouring agents, namely (*s*)-(−)-perillaldehyde and (1*R*)-myrtenal (Uredi *et al.*, 2019 ▸). In C—H activation reactions, the pyridine ring acts as the directing group (Zhang *et al.*, 2014 ▸).

Tieno­pyridine derivatives show diverse pharmacological activities including anti­bacterial activity against a drug-resistant *S. epidermidis* clinical strain (Leal *et al.*, 2008 ▸) and cytotoxic activity against human hepatocellular liver carcinoma (HepG2) (Hassan *et al.*, 2013 ▸) and are used as anti­platelet drugs for the treatment of acute coronary syndromes (Peters *et al.*, 2003 ▸).
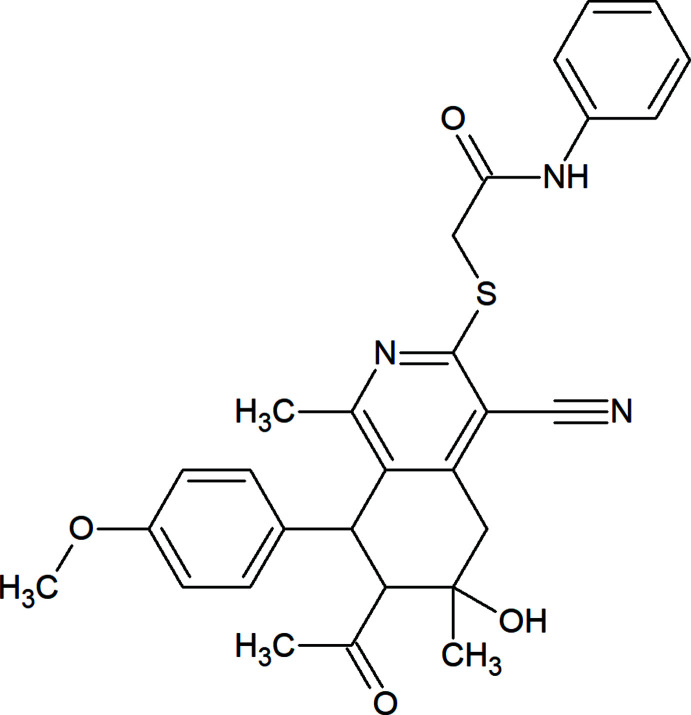


## Structural commentary   

**Figure 1:**
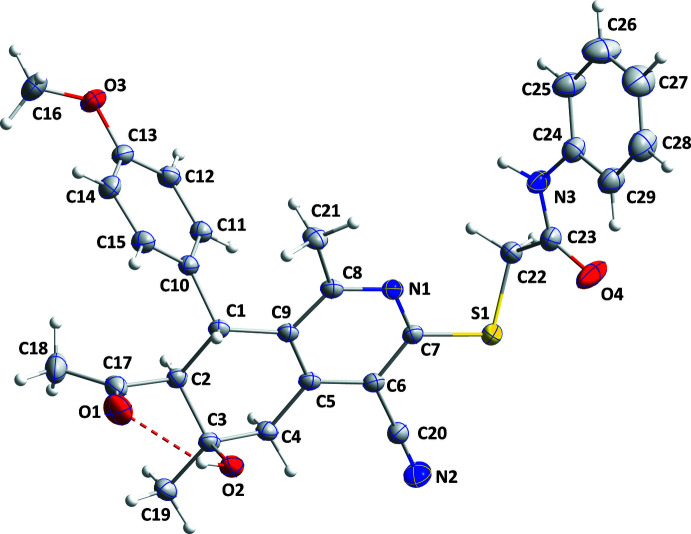
The title mol­ecule with the atom-labelling scheme and 50% probability ellipsoids. The intra­molecular hydrogen bond is depicted by a dashed line.

The title mol­ecule adopts a conformation in which the C24–C29 phenyl group is on the same side of the mean plane of tetra­hydro­iso­quinoline core as the O2–H2*A* hy­droxy group, while the 4-meth­oxy­phenyl group is situated on the opposite side (Fig. 1 ▸). There is an intra­molecular O2—H2*A*⋯O1 hydrogen bond, which controls the orientation of the acetyl group. Puckering analysis (Cremer & Pople, 1975 ▸) shows that the conformation of the C1–C5/C9 ring is close to half-chair with the C2 atom as the flap. The mean planes of the C10–C15 and C24–C29 rings are inclined to that of the pyridine N1/C5–C9 ring by 77.17 (3) and 67.93 (5)°, respectively. All bond lengths and angles appear normal for the given formulation.

## Supra­molecular features   

**Figure 2:**
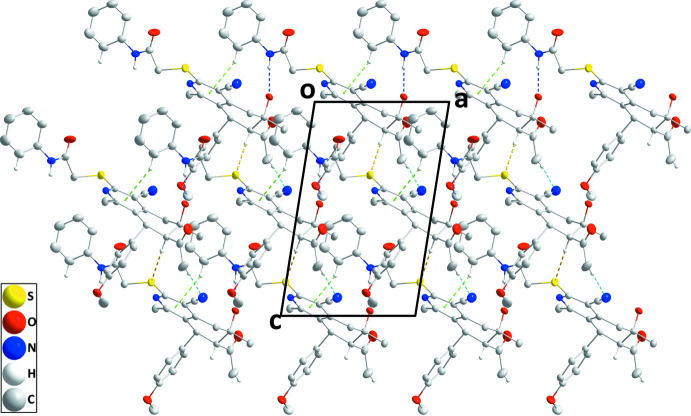
View of a portion of one layer seen along the *b*-axis direction. N—H⋯O, C—H⋯N and C—H⋯S hydrogen bonds are depicted, respectively, by dark-blue, light-blue and yellow dashed lines. C—H⋯π(ring) inter­actions are depicted by green dashed lines.

**Figure 3:**
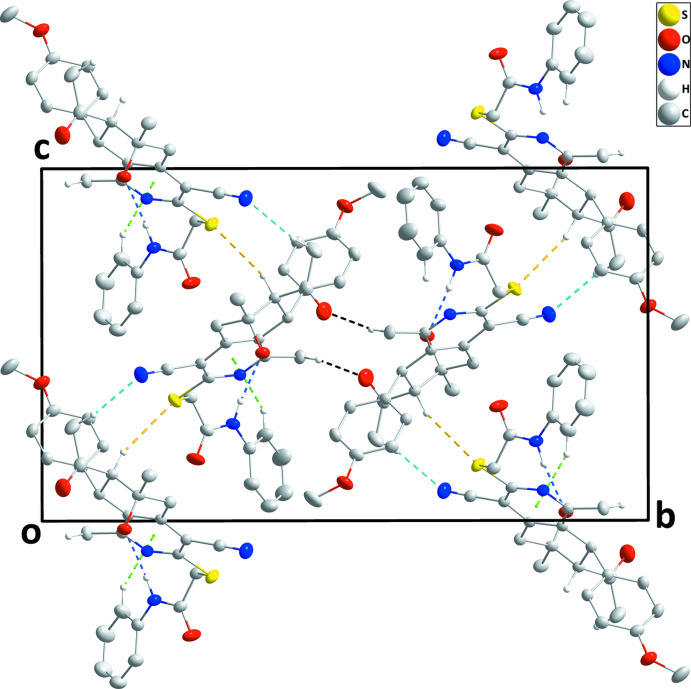
View of portions of two layers showing their connection by C—H⋯O hydrogen bonds (black dashed lines). Other inter­molecular inter­actions are depicted as in Fig. 2 ▸.

In the crystal, N3—H3*A*⋯O2 hydrogen bonds and C25—H25⋯*Cg*1 inter­actions form chains of mol­ecules extending along the *a*-axis direction, which are linked into corrugated layers parallel to (010) by C18—H18*A*⋯N2 and C2—H2⋯S1 hydrogen bonds (Table 1 ▸ and Fig. 2 ▸). The layers are connected by inversion-related C21—H21*C*⋯O1 contacts into the three-dimensional structure (Table 1 ▸ and Fig. 3 ▸).

## Hirshfeld surface analysis   

**Figure 4:**
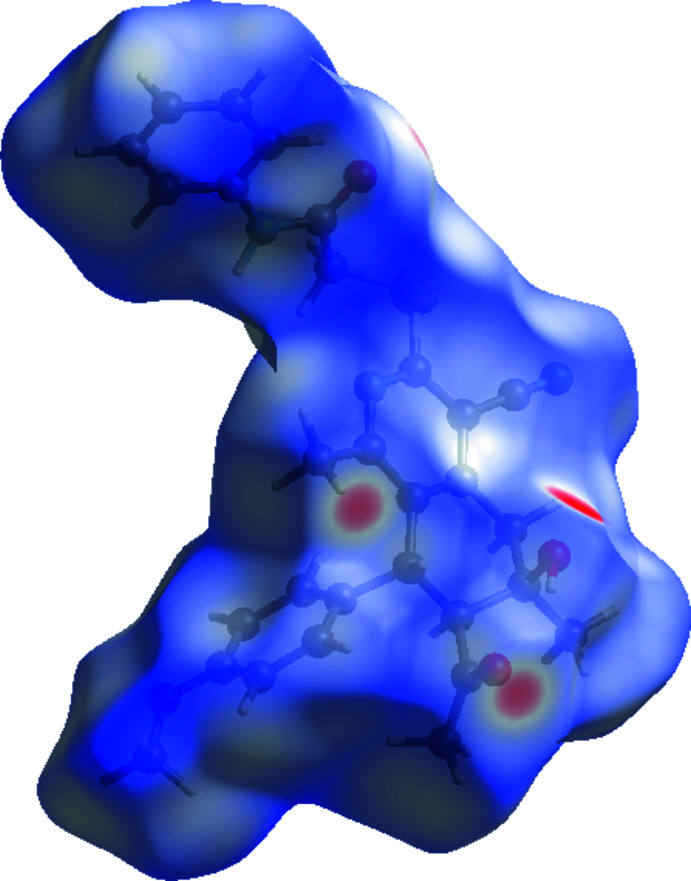
A view of the three-dimensional Hirshfeld surface of the title mol­ecule plotted over *d*_norm_ in the range −0.5236 to +1.6751 a.u.

**Figure 5:**
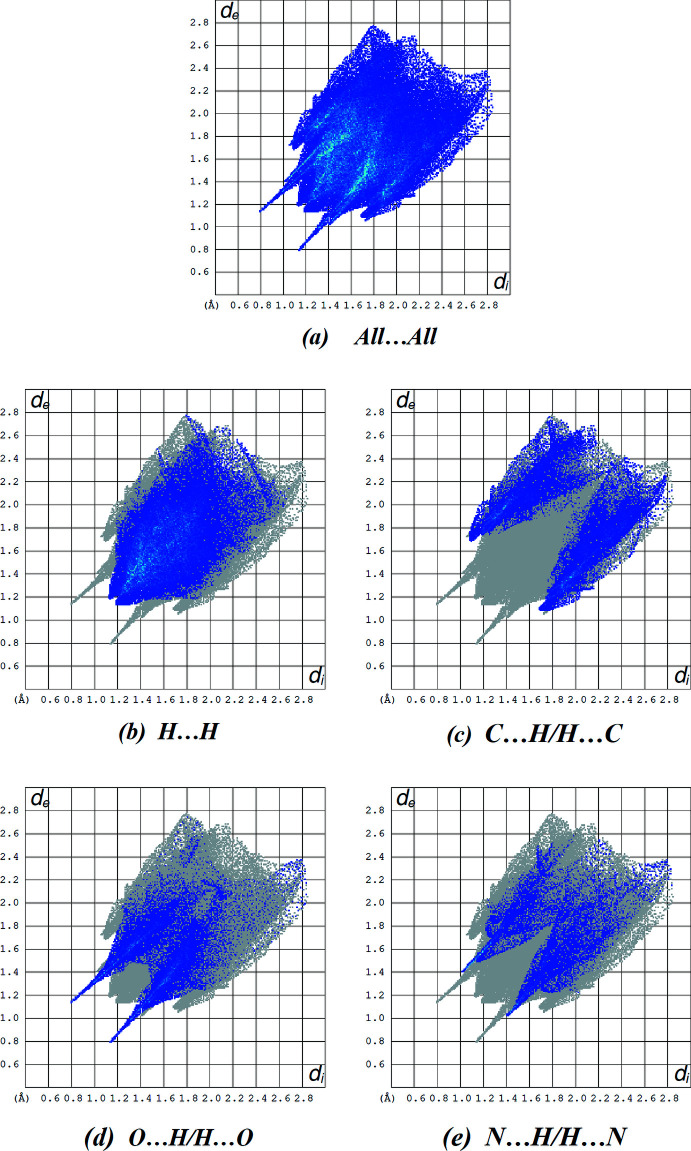
A view of the two-dimensional fingerprint plots for the title compound, showing (*a*) all inter­actions, and those delineated into (*b*) H⋯H, (*c*) C⋯H/H⋯C, (*d*) O⋯H/H⋯O and (*e*) N⋯H/H⋯N inter­actions. The *d*_i_ and *d*_e_ values are the closest inter­nal and external distances (in Å) from given points on the Hirshfeld surface.

Hirshfeld surface calculations (Spackman & Jayatilaka, 2009 ▸) were performed in order to further characterize the supra­molecular association in the title compound. The Hirshfeld surface plotted over *d*_norm_ in the range −0.5236 to +1.6751 a.u. and two-dimensional fingerprint plots (McKinnon *et al.*, 2007 ▸) prepared using *CrystalExplorer 17.5* (Turner *et al.*, 2017 ▸) are shown in Figs. 4 ▸ and 5 ▸, respectively. The red spots on the Hirshfeld surface represent strong inter­molecular inter­actions (Table 2 ▸), whereas the blue colour represents a lack of inter­actions. The fingerprint plots (Fig. 5 ▸) reveal that H⋯H (45.2%), C⋯H/H⋯C (20.2%), O⋯H/H⋯O (15.8%) and N⋯H/H⋯N (11.0%) inter­actions make the greatest contributions to the surface contacts. The lowest contributions are from S⋯H/H⋯S (6.2%), O⋯C/C⋯O (1.2%), N⋯C/C⋯N (0.3%) and C⋯C (0.1%) contacts.

## Database survey   

Nine comparable tetra­hydro­iso­quinoline derivatives are: NAQRIJ (Mague *et al.*, 2017 ▸), KUGLIK (Langenohl *et al.*, 2020 ▸), DUSVIZ (Selvaraj *et al.*, 2020 ▸), AKIVUO (Al-Taifi *et al.*, 2021 ▸), ULUTAZ (Naghiyev *et al.*, 2021 ▸), CARCOQ (Lehmann *et al.*, 2017 ▸), POPYEB (Ben Ali & Retailleau, 2019 ▸), ENOCIU (Naicker *et al.*, 2011 ▸) and NIWPAL (Bouasla *et al.*, 2008 ▸).

In the crystal of NAQRIJ, dimers are formed through complementary sets of inversion-related O—H⋯O and C—H⋯O hydrogen bonds, which are further connected into zigzag chains by pairwise C—H⋯N inter­actions that also form inversion dimers. In KUGLIK, the heterocyclic amines are alternately connected by hydrogen bonds thus forming syndiotactic polymeric chains. The hydrogen-bonding network of water mol­ecules forms planes parallel to (100). In the crystal of DUSVIZ, mol­ecules are linked *via* C—H⋯O hydrogen bonds. For the major disorder component, they form *C*(11) chains that propagate parallel to the *a* axis. In AKIVUO, a layer structure with the layers parallel to (10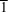) is generated by O—H⋯O and C—H⋯O hydrogen bonds. In ULUTAZ, the mol­ecules are linked *via* N—H⋯O and C—H⋯N hydrogen bonds into a three-dimensional network. Furthermore, the crystal packing is dominated by C—H⋯π contacts involving the phenyl H atoms. In CARCOQ, mol­ecules are linked by an O—H⋯O hydrogen bond, forming chains propagating along the *a*-axis direction. The chains are linked by C—H⋯F hydrogen bonds, forming layers lying parallel to (001). In POPYEB, mol­ecules are packed in a herringbone manner parallel to (103) and (10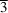) *via* weak C—H⋯O and C—H⋯π(ring) inter­actions. In ENOCIU, various C—H⋯π and C—H⋯O bonds link the mol­ecules together. In NIWPAL, the mol­ecules are linked by N—H⋯O inter­molecular hydrogen bonds involving the sulfonamide function to form an infinite two-dimensional network parallel to (001).

## Synthesis and crystallization   

A mixture of 7-acetyl-4-cyano-1,6-dimethyl-6-hy­droxy-8-(4-meth­oxy­phen­yl)-5,6,7,8-tetra­hydro­iso­quinoline-3(2*H*)-thione (10 mmol), *N*-(phen­yl)-2-chloro­acetamide (10 mmol) and sodium acetate trihydrate (1.77 g, 13 mmol) in ethanol (100 ml) was heated under reflux for 1 h. The reaction mixture was allowed to stand at room temperature overnight. The precipitate that formed was collected and recrystallized from ethanol giving colourless crystals of the title compound, m.p.: 508–510 K, yield 84%. Its IR spectrum showed characteristic absorption bands at 3474 cm^−1^ (OH); 3311 cm^−1^ (NH); 3023 cm^−1^ (C-H aromatic); 2910, 2956 cm^−1^ (C—H aliphatic); 1800, 1900 cm^−1^ (overtones of phenyl group); 2220 cm^−1^ (C≡N) and 1694 cm^−1^ (C=O). Its ^1^H NMR (500 MHz, DMSO-*d_6_*) spectrum exhibited the following signals: δ 10.21 (*s*, 1H, NH), 7.48–7.49 (*d*, 2H, *J* = 5 Hz, Ar-H); 7.22–7.25 (*t*, 2H, Ar-H); 6.97–7.00 (*t*, 1H, Ar-H); 6.89–6.91 (*d*, *J* = 10 Hz, 2H, Ar-H); 6.75–6.77 (*d*, *J* =10 Hz, 2H, Ar-H); 4.84 (*s*, 1H, OH); 4.41–4.43 (*d*, *J* = 10 Hz, 1H, CH at C-8); 4.04–4.11 (*dd*, 2H, SCH_2_); 3.66 (*s*, 3H, OCH_3_); 3.20–3.24 (*d*, *J* = 20 Hz, 1H, CH of cyclo­hexene ring); 2.83–2.85 (*d*, *J* = 10 Hz, 1H, CH at C-7); 2.81–2.84 (*d*, *J* = 15 Hz, 1H, CH of cyclo­hexene ring); 2.08 (*s*, 3H, COCH_3_); 1.86 (*s*, 3H, CH_3_ attached to pyridine ring) and 1.21 (*s*, 3H, CH_3_ at C-6).

## Refinement   

Crystal data, data collection and structure refinement details are summarized in Table 3 ▸. All C-bound H atoms were placed in geometrically idealized positions (C—H = 0.95–1.00 Å) while those attached to O and N atoms were positioned from a difference map, refined for a few cycles to ensure that reasonable displacement parameters could be achieved, and then their coordinates were adjusted to give O—H = 0.87 and N—H = 0.91 Å. All H atoms were refined using a riding model with isotropic displacement parameters 1.2–1.5 times those of the parent atoms.

## Supplementary Material

Crystal structure: contains datablock(s) I, global. DOI: 10.1107/S2056989021005430/yk2151sup1.cif

Structure factors: contains datablock(s) I. DOI: 10.1107/S2056989021005430/yk2151Isup2.hkl

Supporting information file. DOI: 10.1107/S2056989021005430/yk2151Isup3.cml

CCDC reference: 2085564

Additional supporting information:  crystallographic information; 3D view; checkCIF report

## Figures and Tables

**Table 1:** Hydrogen-bond geometry (Å, °) *Cg*1 is the centroid of the N1/C–C9 ring.

*D*—H⋯*A*	*D*—H	H⋯*A*	*D*⋯*A*	*D*—H⋯*A*
O2—H2*A*⋯O1	0.87	2.13	2.8343 (14)	138
N3—H3*A*⋯O2^i^	0.91	2.03	2.9335 (14)	174
C2—H2⋯S1^ii^	1.00	2.86	3.8269 (12)	163
C18—H18*A*⋯N2^ii^	0.98	2.52	3.493 (2)	170
C21—H21*C*⋯O1^iii^	0.98	2.39	3.3593 (16)	169
C25—H25⋯*Cg*1^i^	0.95	2.79	3.6141 (18)	146

Symmetry codes: (i) x-1, y, z; (ii) x+{\script{1\over 2}}, -y+{\script{1\over 2}}, z+{\script{1\over 2}}; (iii) -x+2, -y+1, -z+1.

**Table 2:** Summary of short inter­molecular contacts (Å) in the title structure

Contact	Distance	Symmetry operation
N2⋯H18*A*	2.52	−{1\over 2} + *x*, {1\over 2} − *y*, − {1\over 2} + *z*
H21*C*⋯O1	2.39	2 − *x*, 1 − *y*, 1 − *z*
O2⋯H3*A*	2.03	1 + *x*, *y*, *z*
O3⋯H27	2.70	1 + *x*, *y*, 1 + *z*
N2⋯H16*B*	2.69	{3\over 2} − *x*, − {1\over 2} + *y*, {3\over 2} − *z*
N2⋯H12	2.61	{1\over 2} + *x*, {1\over 2} − *y*, −{1\over 2} + *z*
H26⋯H18*C*	2.49	1 − *x*, 1 − *y*, 1 − *z*
H27⋯H16*B*	2.47	−*x*, 1 − *y*, 1 − *z*

**Table 3:** Experimental details

Crystal data	
Chemical formula	C_29_H_29_N_3_O_4_S
*M*_r_	515.61
Crystal system, space group	Monoclinic, *P*2_1_/*n*
Temperature (K)	150
*a*, *b*, *c* (Å)	8.4506 (16), 23.112 (5), 13.601 (3)
β (°)	99.021 (3)
*V* (Å^3^)	2623.5 (9)
*Z*	4
Radiation type	Mo *K*α
μ (mm^−1^)	0.16
Crystal size (mm)	0.39 × 0.25 × 0.14

Data collection	
Diffractometer	Bruker *SMART* *APEX* CCD
Absorption correction	Multi-scan (*SADABS*; Krause *et al.*, 2015 ▸)
*T*_min_, *T*_max_	0.89, 0.98
No. of measured, independent and observed [*I* > 2σ(*I*)] reflections	50421, 7039, 5632
*R*_int_	0.034
(sin θ/λ)_max_ (Å^−1^)	0.684

Refinement	
*R*[*F* ^2^ > 2σ(*F* ^2^)], *wR*(*F* ^2^), *S*	0.043, 0.126, 1.10
No. of reflections	7039
No. of parameters	338
H-atom treatment	H-atom parameters constrained
Δρ_max_, Δρ_min_ (e Å^−3^)	0.49, −0.20

Computer programs: *APEX3* (Bruker, 2016 ▸), *SAINT* (Bruker, 2016 ▸), *SHELXT* (Sheldrick, 2015*a* ▸), *SHELXL2018/1* (Sheldrick, 2015*b* ▸), *DIAMOND* (Brandenburg & Putz, 2012 ▸), *SHELXTL* (Sheldrick, 2008 ▸).

## supplementary crystallographic information

### Crystal data

**Table:**

C_29_H_29_N_3_O_4_S	*F*(000) = 1088
*M**_r_* = 515.61	*D*_x_ = 1.305 Mg m^−^^3^
Monoclinic, *P*2_1_/*n*	Mo *K*α radiation, λ = 0.71073 Å
*a* = 8.4506 (16) Å	Cell parameters from 9458 reflections
*b* = 23.112 (5) Å	θ = 2.3–29.1°
*c* = 13.601 (3) Å	µ = 0.16 mm^−^^1^
β = 99.021 (3)°	*T* = 150 K
*V* = 2623.5 (9) Å^3^	Block, colourless
*Z* = 4	0.39 × 0.25 × 0.14 mm

### Data collection

**Table:**

Bruker SMART APEX CCD diffractometer	7039 independent reflections
Radiation source: fine-focus sealed tube	5632 reflections with *I* > 2σ(*I*)
Graphite monochromator	*R*_int_ = 0.034
Detector resolution: 8.3333 pixels mm^-1^	θ_max_ = 29.1°, θ_min_ = 1.8°
φ and ω scans	*h* = −11→11
Absorption correction: multi-scan (*SADABS*; Krause *et al.*, 2015)	*k* = −31→31
*T*_min_ = 0.89, *T*_max_ = 0.98	*l* = −18→18
50421 measured reflections	

### Refinement

**Table:**

Refinement on *F*^2^	Primary atom site location: dual
Least-squares matrix: full	Secondary atom site location: difference Fourier map
*R*[*F*^2^ > 2σ(*F*^2^)] = 0.043	Hydrogen site location: mixed
*wR*(*F*^2^) = 0.126	H-atom parameters constrained
*S* = 1.10	*w* = 1/[σ^2^(*F*_o_^2^) + (0.0798*P*)^2^ + 0.1546*P*] where *P* = (*F*_o_^2^ + 2*F*_c_^2^)/3
7039 reflections	(Δ/σ)_max_ = 0.001
338 parameters	Δρ_max_ = 0.49 e Å^−^^3^
0 restraints	Δρ_min_ = −0.20 e Å^−^^3^

### Special details

**Table:**

Experimental. The diffraction data were obtained from 3 sets of 400 frames, each of width 0.5° in ω, collected at φ = 0.00, 90.00 and 180.00° and 2 sets of 800 frames, each of width 0.45° in φ, collected at ω = –30.00 and 210.00°. The scan time was 20 sec/frame.
Geometry. All esds (except the esd in the dihedral angle between two l.s. planes) are estimated using the full covariance matrix. The cell esds are taken into account individually in the estimation of esds in distances, angles and torsion angles; correlations between esds in cell parameters are only used when they are defined by crystal symmetry. An approximate (isotropic) treatment of cell esds is used for estimating esds involving l.s. planes.
Refinement. Refinement of F^2^ against ALL reflections. The weighted R-factor wR and goodness of fit S are based on F^2^, conventional R-factors R are based on F, with F set to zero for negative F^2^. The threshold expression of F^2^ > 2sigma(F^2^) is used only for calculating R-factors(gt) etc. and is not relevant to the choice of reflections for refinement. R-factors based on F^2^ are statistically about twice as large as those based on F, and R- factors based on ALL data will be even larger. H-atoms attached to carbon were placed in calculated positions (C—H = 0.95 - 1.00 Å) while that attached to oxygen was placed in a location derived from a difference map and its coordinates adjusted to give O—H = 0.87 %A. All were included as riding contributions with isotropic displacement parameters 1.2 - 1.5 times those of the attached atoms.

### Fractional atomic coordinates and isotropic or equivalent isotropic displacement parameters (Å^2^)

**Table:**

	*x*	*y*	*z*	*U*_iso_*/*U*_eq_	
S1	0.49743 (4)	0.21951 (2)	0.34201 (2)	0.02816 (10)	
O1	1.20833 (12)	0.46549 (4)	0.59462 (8)	0.0424 (3)	
O2	1.15692 (10)	0.36411 (4)	0.47667 (6)	0.02629 (19)	
H2A	1.193331	0.399275	0.484612	0.039*	
O3	0.60807 (11)	0.50037 (4)	0.89281 (7)	0.0355 (2)	
O4	0.22412 (14)	0.25356 (5)	0.17522 (7)	0.0472 (3)	
N1	0.55129 (11)	0.32668 (4)	0.41455 (7)	0.0221 (2)	
N2	0.89889 (15)	0.16351 (5)	0.41504 (9)	0.0376 (3)	
N3	0.11079 (13)	0.31512 (5)	0.27523 (7)	0.0283 (2)	
H3A	0.123005	0.327808	0.339295	0.034*	
C1	0.89205 (13)	0.40578 (4)	0.57616 (8)	0.0193 (2)	
H1	0.912963	0.436858	0.528669	0.023*	
C2	1.05704 (13)	0.38474 (5)	0.63229 (8)	0.0205 (2)	
H2	1.037934	0.365533	0.695266	0.025*	
C3	1.14103 (13)	0.34073 (5)	0.57288 (8)	0.0216 (2)	
C4	1.03246 (13)	0.28858 (5)	0.55317 (9)	0.0225 (2)	
H4A	1.079572	0.261207	0.509824	0.027*	
H4B	1.026798	0.268725	0.617023	0.027*	
C5	0.86588 (13)	0.30393 (5)	0.50451 (8)	0.0193 (2)	
C6	0.77354 (13)	0.26220 (5)	0.44664 (8)	0.0204 (2)	
C7	0.61510 (13)	0.27491 (5)	0.40627 (8)	0.0208 (2)	
C8	0.64077 (13)	0.36804 (5)	0.46651 (8)	0.0207 (2)	
C9	0.79759 (13)	0.35800 (4)	0.51612 (8)	0.0187 (2)	
C10	0.80553 (13)	0.43324 (5)	0.65445 (8)	0.0204 (2)	
C11	0.71854 (14)	0.39936 (5)	0.71205 (9)	0.0225 (2)	
H11	0.704102	0.359333	0.697472	0.027*	
C12	0.65289 (14)	0.42303 (5)	0.79006 (9)	0.0251 (2)	
H12	0.592213	0.399454	0.827603	0.030*	
C13	0.67580 (14)	0.48133 (5)	0.81346 (9)	0.0263 (2)	
C14	0.76132 (15)	0.51598 (5)	0.75698 (9)	0.0284 (3)	
H14	0.776837	0.555878	0.772287	0.034*	
C15	0.82427 (14)	0.49165 (5)	0.67751 (9)	0.0249 (2)	
H15	0.881237	0.515577	0.638293	0.030*	
C16	0.6676 (2)	0.55312 (7)	0.93842 (12)	0.0465 (4)	
H16A	0.784465	0.550913	0.955575	0.070*	
H16B	0.620648	0.559492	0.999005	0.070*	
H16C	0.638604	0.585256	0.892094	0.070*	
C17	1.16474 (14)	0.43673 (5)	0.66046 (10)	0.0286 (3)	
C18	1.21626 (19)	0.44933 (7)	0.76823 (11)	0.0431 (4)	
H18A	1.267619	0.414967	0.801321	0.065*	
H18B	1.122528	0.459723	0.798737	0.065*	
H18C	1.292503	0.481576	0.775466	0.065*	
C19	1.30468 (14)	0.32302 (6)	0.62869 (9)	0.0290 (3)	
H19A	1.293501	0.310103	0.695891	0.044*	
H19B	1.377685	0.356189	0.633079	0.044*	
H19C	1.347991	0.291385	0.592961	0.044*	
C20	0.84162 (14)	0.20690 (5)	0.43026 (9)	0.0247 (2)	
C21	0.56021 (15)	0.42595 (5)	0.46529 (10)	0.0265 (2)	
H21A	0.519924	0.431841	0.528302	0.040*	
H21B	0.470632	0.427392	0.410039	0.040*	
H21C	0.637465	0.456448	0.456723	0.040*	
C22	0.29909 (14)	0.24507 (5)	0.35184 (9)	0.0257 (2)	
H22A	0.234973	0.212181	0.370639	0.031*	
H22B	0.307145	0.274003	0.406068	0.031*	
C23	0.21135 (15)	0.27208 (5)	0.25746 (9)	0.0273 (3)	
C24	−0.00831 (16)	0.34254 (5)	0.20589 (9)	0.0285 (3)	
C25	−0.13620 (19)	0.36782 (6)	0.24298 (11)	0.0396 (3)	
H25	−0.141122	0.365874	0.312204	0.047*	
C26	−0.2556 (2)	0.39563 (8)	0.18009 (13)	0.0514 (4)	
H26	−0.343050	0.412373	0.206057	0.062*	
C27	−0.2491 (2)	0.39938 (7)	0.07906 (12)	0.0492 (4)	
H27	−0.331596	0.418599	0.035625	0.059*	
C28	−0.1216 (2)	0.37489 (7)	0.04246 (11)	0.0437 (4)	
H28	−0.115917	0.377920	−0.026552	0.052*	
C29	−0.00134 (18)	0.34587 (6)	0.10445 (10)	0.0351 (3)	
H29	0.084748	0.328480	0.077982	0.042*	

### Atomic displacement parameters (Å^2^)

**Table:**

	*U*^11^	*U*^22^	*U*^33^	*U*^12^	*U*^13^	*U*^23^
S1	0.02272 (16)	0.03016 (17)	0.03103 (18)	0.00082 (11)	0.00246 (12)	−0.01154 (12)
O1	0.0374 (5)	0.0381 (5)	0.0533 (6)	−0.0143 (4)	0.0119 (5)	0.0018 (5)
O2	0.0251 (4)	0.0342 (4)	0.0211 (4)	−0.0026 (3)	0.0085 (3)	0.0007 (3)
O3	0.0362 (5)	0.0424 (5)	0.0303 (5)	−0.0006 (4)	0.0130 (4)	−0.0128 (4)
O4	0.0612 (7)	0.0552 (6)	0.0236 (5)	0.0261 (5)	0.0014 (5)	−0.0100 (4)
N1	0.0203 (5)	0.0248 (4)	0.0216 (5)	0.0017 (4)	0.0043 (4)	−0.0006 (4)
N2	0.0387 (6)	0.0308 (5)	0.0423 (7)	0.0087 (5)	0.0035 (5)	−0.0053 (5)
N3	0.0318 (6)	0.0322 (5)	0.0209 (5)	0.0055 (4)	0.0039 (4)	−0.0030 (4)
C1	0.0191 (5)	0.0201 (5)	0.0194 (5)	−0.0001 (4)	0.0051 (4)	0.0011 (4)
C2	0.0203 (5)	0.0231 (5)	0.0186 (5)	−0.0001 (4)	0.0044 (4)	0.0003 (4)
C3	0.0193 (5)	0.0275 (5)	0.0186 (5)	0.0013 (4)	0.0048 (4)	0.0005 (4)
C4	0.0193 (5)	0.0238 (5)	0.0244 (6)	0.0038 (4)	0.0034 (4)	−0.0004 (4)
C5	0.0186 (5)	0.0222 (5)	0.0181 (5)	0.0013 (4)	0.0061 (4)	0.0019 (4)
C6	0.0209 (5)	0.0215 (5)	0.0196 (5)	0.0024 (4)	0.0054 (4)	−0.0002 (4)
C7	0.0199 (5)	0.0244 (5)	0.0188 (5)	−0.0002 (4)	0.0052 (4)	−0.0015 (4)
C8	0.0202 (5)	0.0222 (5)	0.0206 (5)	0.0016 (4)	0.0058 (4)	0.0015 (4)
C9	0.0183 (5)	0.0206 (5)	0.0180 (5)	0.0006 (4)	0.0054 (4)	0.0014 (4)
C10	0.0194 (5)	0.0209 (5)	0.0210 (5)	0.0015 (4)	0.0034 (4)	−0.0004 (4)
C11	0.0222 (5)	0.0216 (5)	0.0238 (5)	0.0002 (4)	0.0046 (4)	0.0005 (4)
C12	0.0224 (6)	0.0291 (6)	0.0247 (6)	−0.0012 (4)	0.0065 (5)	0.0006 (4)
C13	0.0222 (6)	0.0334 (6)	0.0237 (6)	0.0037 (5)	0.0045 (4)	−0.0058 (5)
C14	0.0297 (6)	0.0234 (5)	0.0319 (6)	0.0006 (5)	0.0049 (5)	−0.0058 (5)
C15	0.0250 (6)	0.0219 (5)	0.0287 (6)	−0.0007 (4)	0.0074 (5)	0.0010 (4)
C16	0.0458 (9)	0.0524 (9)	0.0426 (8)	−0.0003 (7)	0.0107 (7)	−0.0253 (7)
C17	0.0217 (6)	0.0272 (6)	0.0359 (7)	0.0009 (4)	0.0016 (5)	−0.0037 (5)
C18	0.0401 (8)	0.0420 (8)	0.0420 (8)	0.0037 (6)	−0.0101 (6)	−0.0156 (6)
C19	0.0202 (6)	0.0375 (7)	0.0285 (6)	0.0038 (5)	0.0011 (5)	−0.0028 (5)
C20	0.0243 (6)	0.0260 (5)	0.0236 (6)	0.0017 (4)	0.0030 (5)	−0.0023 (4)
C21	0.0230 (6)	0.0220 (5)	0.0335 (6)	0.0044 (4)	0.0017 (5)	0.0026 (5)
C22	0.0217 (5)	0.0305 (6)	0.0248 (6)	−0.0004 (5)	0.0035 (4)	−0.0037 (5)
C23	0.0275 (6)	0.0301 (6)	0.0236 (6)	0.0000 (5)	0.0026 (5)	−0.0054 (5)
C24	0.0332 (7)	0.0260 (6)	0.0261 (6)	0.0022 (5)	0.0037 (5)	−0.0003 (5)
C25	0.0448 (8)	0.0434 (7)	0.0323 (7)	0.0137 (6)	0.0118 (6)	0.0066 (6)
C26	0.0488 (9)	0.0588 (10)	0.0482 (9)	0.0255 (8)	0.0126 (8)	0.0086 (7)
C27	0.0509 (10)	0.0524 (9)	0.0413 (8)	0.0168 (7)	−0.0023 (7)	0.0072 (7)
C28	0.0532 (9)	0.0480 (8)	0.0277 (7)	0.0080 (7)	−0.0006 (6)	0.0016 (6)
C29	0.0394 (7)	0.0394 (7)	0.0264 (6)	0.0052 (6)	0.0052 (6)	−0.0020 (5)

### Geometric parameters (Å, º)

**Table:**

S1—C7	1.7661 (12)	C11—H11	0.9500
S1—C22	1.8018 (12)	C12—C13	1.3911 (17)
O1—C17	1.2176 (16)	C12—H12	0.9500
O2—C3	1.4414 (13)	C13—C14	1.3884 (18)
O2—H2A	0.8696	C14—C15	1.3955 (16)
O3—C13	1.3711 (14)	C14—H14	0.9500
O3—C16	1.4233 (17)	C15—H15	0.9500
O4—C23	1.2182 (15)	C16—H16A	0.9800
N1—C7	1.3245 (14)	C16—H16B	0.9800
N1—C8	1.3482 (15)	C16—H16C	0.9800
N2—C20	1.1463 (15)	C17—C18	1.4908 (19)
N3—C23	1.3545 (16)	C18—H18A	0.9800
N3—C24	1.4164 (16)	C18—H18B	0.9800
N3—H3A	0.9097	C18—H18C	0.9800
C1—C10	1.5216 (15)	C19—H19A	0.9800
C1—C9	1.5229 (15)	C19—H19B	0.9800
C1—C2	1.5587 (15)	C19—H19C	0.9800
C1—H1	1.0000	C21—H21A	0.9800
C2—C17	1.5198 (16)	C21—H21B	0.9800
C2—C3	1.5399 (15)	C21—H21C	0.9800
C2—H2	1.0000	C22—C23	1.5128 (17)
C3—C4	1.5129 (16)	C22—H22A	0.9900
C3—C19	1.5255 (16)	C22—H22B	0.9900
C4—C5	1.5011 (15)	C24—C25	1.3911 (19)
C4—H4A	0.9900	C24—C29	1.3923 (18)
C4—H4B	0.9900	C25—C26	1.375 (2)
C5—C9	1.3958 (15)	C25—H25	0.9500
C5—C6	1.4021 (16)	C26—C27	1.386 (2)
C6—C7	1.3964 (16)	C26—H26	0.9500
C6—C20	1.4334 (15)	C27—C28	1.378 (2)
C8—C9	1.4088 (15)	C27—H27	0.9500
C8—C21	1.5006 (15)	C28—C29	1.387 (2)
C10—C15	1.3894 (16)	C28—H28	0.9500
C10—C11	1.3957 (15)	C29—H29	0.9500
C11—C12	1.3847 (16)		
			
C7—S1—C22	100.59 (6)	C15—C14—H14	120.3
C3—O2—H2A	108.6	C10—C15—C14	121.50 (11)
C13—O3—C16	117.10 (11)	C10—C15—H15	119.3
C7—N1—C8	118.87 (10)	C14—C15—H15	119.3
C23—N3—C24	127.59 (10)	O3—C16—H16A	109.5
C23—N3—H3A	115.3	O3—C16—H16B	109.5
C24—N3—H3A	117.1	H16A—C16—H16B	109.5
C10—C1—C9	114.12 (9)	O3—C16—H16C	109.5
C10—C1—C2	106.17 (9)	H16A—C16—H16C	109.5
C9—C1—C2	112.94 (8)	H16B—C16—H16C	109.5
C10—C1—H1	107.8	O1—C17—C18	122.75 (13)
C9—C1—H1	107.8	O1—C17—C2	118.98 (12)
C2—C1—H1	107.8	C18—C17—C2	118.24 (12)
C17—C2—C3	110.33 (9)	C17—C18—H18A	109.5
C17—C2—C1	109.33 (9)	C17—C18—H18B	109.5
C3—C2—C1	113.55 (9)	H18A—C18—H18B	109.5
C17—C2—H2	107.8	C17—C18—H18C	109.5
C3—C2—H2	107.8	H18A—C18—H18C	109.5
C1—C2—H2	107.8	H18B—C18—H18C	109.5
O2—C3—C4	106.15 (9)	C3—C19—H19A	109.5
O2—C3—C19	110.25 (9)	C3—C19—H19B	109.5
C4—C3—C19	110.59 (10)	H19A—C19—H19B	109.5
O2—C3—C2	110.17 (9)	C3—C19—H19C	109.5
C4—C3—C2	107.61 (9)	H19A—C19—H19C	109.5
C19—C3—C2	111.88 (9)	H19B—C19—H19C	109.5
C5—C4—C3	112.99 (9)	N2—C20—C6	177.76 (14)
C5—C4—H4A	109.0	C8—C21—H21A	109.5
C3—C4—H4A	109.0	C8—C21—H21B	109.5
C5—C4—H4B	109.0	H21A—C21—H21B	109.5
C3—C4—H4B	109.0	C8—C21—H21C	109.5
H4A—C4—H4B	107.8	H21A—C21—H21C	109.5
C9—C5—C6	118.41 (10)	H21B—C21—H21C	109.5
C9—C5—C4	122.51 (10)	C23—C22—S1	114.22 (9)
C6—C5—C4	119.07 (9)	C23—C22—H22A	108.7
C7—C6—C5	119.41 (10)	S1—C22—H22A	108.7
C7—C6—C20	120.66 (10)	C23—C22—H22B	108.7
C5—C6—C20	119.93 (10)	S1—C22—H22B	108.7
N1—C7—C6	122.32 (10)	H22A—C22—H22B	107.6
N1—C7—S1	119.46 (9)	O4—C23—N3	124.77 (12)
C6—C7—S1	118.22 (8)	O4—C23—C22	122.14 (11)
N1—C8—C9	122.85 (10)	N3—C23—C22	112.92 (10)
N1—C8—C21	114.24 (10)	C25—C24—C29	119.47 (12)
C9—C8—C21	122.90 (10)	C25—C24—N3	117.25 (11)
C5—C9—C8	117.89 (10)	C29—C24—N3	123.27 (12)
C5—C9—C1	121.24 (10)	C26—C25—C24	120.45 (14)
C8—C9—C1	120.80 (9)	C26—C25—H25	119.8
C15—C10—C11	118.02 (10)	C24—C25—H25	119.8
C15—C10—C1	120.86 (10)	C25—C26—C27	120.43 (14)
C11—C10—C1	120.80 (10)	C25—C26—H26	119.8
C12—C11—C10	121.20 (11)	C27—C26—H26	119.8
C12—C11—H11	119.4	C28—C27—C26	119.13 (14)
C10—C11—H11	119.4	C28—C27—H27	120.4
C11—C12—C13	119.99 (11)	C26—C27—H27	120.4
C11—C12—H12	120.0	C27—C28—C29	121.28 (14)
C13—C12—H12	120.0	C27—C28—H28	119.4
O3—C13—C14	124.61 (11)	C29—C28—H28	119.4
O3—C13—C12	115.51 (11)	C28—C29—C24	119.22 (13)
C14—C13—C12	119.87 (11)	C28—C29—H29	120.4
C13—C14—C15	119.40 (11)	C24—C29—H29	120.4
C13—C14—H14	120.3		
			
C10—C1—C2—C17	−73.07 (11)	C2—C1—C9—C5	−7.81 (14)
C9—C1—C2—C17	161.14 (9)	C10—C1—C9—C8	53.90 (13)
C10—C1—C2—C3	163.24 (9)	C2—C1—C9—C8	175.28 (9)
C9—C1—C2—C3	37.45 (12)	C9—C1—C10—C15	−145.75 (11)
C17—C2—C3—O2	−68.34 (12)	C2—C1—C10—C15	89.18 (12)
C1—C2—C3—O2	54.80 (12)	C9—C1—C10—C11	40.85 (14)
C17—C2—C3—C4	176.35 (9)	C2—C1—C10—C11	−84.22 (12)
C1—C2—C3—C4	−60.51 (11)	C15—C10—C11—C12	−0.12 (17)
C17—C2—C3—C19	54.68 (13)	C1—C10—C11—C12	173.46 (10)
C1—C2—C3—C19	177.82 (9)	C10—C11—C12—C13	−1.27 (18)
O2—C3—C4—C5	−64.32 (11)	C16—O3—C13—C14	−19.52 (18)
C19—C3—C4—C5	176.09 (9)	C16—O3—C13—C12	161.09 (12)
C2—C3—C4—C5	53.62 (12)	C11—C12—C13—O3	−179.06 (10)
C3—C4—C5—C9	−26.75 (15)	C11—C12—C13—C14	1.52 (18)
C3—C4—C5—C6	154.50 (10)	O3—C13—C14—C15	−179.75 (11)
C9—C5—C6—C7	−2.77 (16)	C12—C13—C14—C15	−0.39 (18)
C4—C5—C6—C7	176.02 (10)	C11—C10—C15—C14	1.28 (17)
C9—C5—C6—C20	177.84 (10)	C1—C10—C15—C14	−172.30 (11)
C4—C5—C6—C20	−3.36 (16)	C13—C14—C15—C10	−1.03 (18)
C8—N1—C7—C6	−2.18 (16)	C3—C2—C17—O1	59.92 (14)
C8—N1—C7—S1	177.89 (8)	C1—C2—C17—O1	−65.65 (14)
C5—C6—C7—N1	4.95 (17)	C3—C2—C17—C18	−118.40 (12)
C20—C6—C7—N1	−175.67 (11)	C1—C2—C17—C18	116.04 (12)
C5—C6—C7—S1	−175.12 (8)	C7—S1—C22—C23	103.44 (9)
C20—C6—C7—S1	4.25 (15)	C24—N3—C23—O4	7.0 (2)
C22—S1—C7—N1	−22.84 (10)	C24—N3—C23—C22	−168.25 (11)
C22—S1—C7—C6	157.23 (9)	S1—C22—C23—O4	36.74 (17)
C7—N1—C8—C9	−2.71 (16)	S1—C22—C23—N3	−147.86 (9)
C7—N1—C8—C21	176.15 (10)	C23—N3—C24—C25	155.07 (13)
C6—C5—C9—C8	−1.73 (15)	C23—N3—C24—C29	−25.9 (2)
C4—C5—C9—C8	179.52 (10)	C29—C24—C25—C26	0.4 (2)
C6—C5—C9—C1	−178.72 (9)	N3—C24—C25—C26	179.45 (14)
C4—C5—C9—C1	2.53 (16)	C24—C25—C26—C27	−0.7 (3)
N1—C8—C9—C5	4.65 (16)	C25—C26—C27—C28	0.0 (3)
C21—C8—C9—C5	−174.11 (10)	C26—C27—C28—C29	1.0 (3)
N1—C8—C9—C1	−178.34 (10)	C27—C28—C29—C24	−1.3 (2)
C21—C8—C9—C1	2.90 (16)	C25—C24—C29—C28	0.6 (2)
C10—C1—C9—C5	−129.19 (11)	N3—C24—C29—C28	−178.39 (13)

### Hydrogen-bond geometry (Å, º)

*Cg*1 is the centroid of the N1/C–C9 ring.

**Table:**

*D*—H···*A*	*D*—H	H···*A*	*D*···*A*	*D*—H···*A*
O2—H2*A*···O1	0.87	2.13	2.8343 (14)	138
N3—H3*A*···O2^i^	0.91	2.03	2.9335 (14)	174
C2—H2···S1^ii^	1.00	2.86	3.8269 (12)	163
C18—H18*A*···N2^ii^	0.98	2.52	3.493 (2)	170
C21—H21*C*···O1^iii^	0.98	2.39	3.3593 (16)	169
C25—H25···*Cg*1^i^	0.95	2.79	3.6141 (18)	146

Symmetry codes: (i) *x*−1, *y*, *z*; (ii) *x*+1/2, −*y*+1/2, *z*+1/2; (iii) −*x*+2, −*y*+1, −*z*+1.
